# Tumor Inhibitory Effect of IRCR201, a Novel Cross-Reactive c-Met Antibody Targeting the PSI Domain

**DOI:** 10.3390/ijms18091968

**Published:** 2017-09-13

**Authors:** Hyunkyu Park, Donggeon Kim, Eunmi Kim, Jason K. Sa, Hee Won Lee, Suji Yu, Jiwon Oh, Seok-Hyung Kim, Yeup Yoon, Do-Hyun Nam

**Affiliations:** 1 Department of Health Sciences and Technology, Samsung Advanced Institute for Health Sciences and Technology, Sungkyunkwan University, Seoul 06351, Korea; parkhk0427@skku.edu (H.P.); iammirlee@skku.edu (H.W.L.); parmenides.kim@samsung.com (S.-H.K.); 2 Institute for Refractory Cancer Research, Research Institute for Future Medicine, Samsung Medical Center, Seoul 06351, Korea; donggeon.kim@gmail.com (D.K.); lovely8925@gmail.com (E.K.); js2dark@gmail.com (J.K.S.); yoosuji0816@gmail.com (S.Y.); wpqkf2228601@gmail.com (J.O.); 3 Department of Pathology, Samsung Medical Center, Sungkyunkwan University School of Medicine, Seoul 06351, Korea; 4 Department of Neurosurgery, Samsung Medical Center, Sungkyunkwan University School of Medicine, Seoul 06351, Korea

**Keywords:** IRCR201, fully human antibody, cancer, c-Met, PSI domain, cross-reactivity

## Abstract

Hepatocyte growth factor receptor (HGFR, c-Met) is an essential member of the receptor tyrosine kinase (RTK) family that is often dysregulated during tumor progression, driving a malignant phenotypic state and modulating important cellular functions including tumor growth, invasion, metastasis, and angiogenesis, providing a strong rationale for targeting HGF/c-Met signaling axis in cancer therapy. Based on its protumorigenic potentials, we developed IRCR201, a potent antagonistic antibody targeting the plexin-semaphorin-integrin (PSI) domain of c-Met, using synthetic human antibody phage libraries. We characterized and evaluated the biochemical properties and tumor inhibitory effect of IRCR201 in vitro and in vivo. IRCR201 is a novel fully-human bivalent therapeutic antibody that exhibits cross-reactivity against both human and mouse c-Met proteins with high affinity and specificity. IRCR201 displayed low agonist activity and rapidly depleted total c-Met protein via the lysosomal degradation pathway, inhibiting c-Met-dependent downstream activation and attenuating cellular proliferation in various c-Met-expressing cancer cells. In vivo tumor xenograft models also demonstrated the superior tumor inhibitory responsiveness of IRCR201. Taken together, IRCR201 provides a promising therapeutic agent for c-Met-positive cancer patients through suppressing the c-Met signaling pathway and tumor growth.

## 1. Introduction

Aberrant activation of c-Met, a receptor tyrosine kinase, is frequently observed in various cancer types via a hepatocyte growth factor (HGF)-dependent or -independent manner [1,2,3,4]. c-Met activation via its ligand, HGF, promotes various tumorigenic potentials including malignant progression, angiogenesis, mesenchymal–epithelial transition (MET), invasion, and metastasis [5,6,7]. In addition to HGF-induced c-Met activation, the c-Met pathway is also stimulated through HGF-independent mechanisms such as c-Met transcriptional upregulation, genomic amplifications, mutations, and structural variations including gene fusions [1,2]. c-Met amplification and constitutive kinase activation have been reported in a number of human primary tumors including gastric cancer and non-small cell lung cancer (NSCLC) [8,9]. Moreover, elevated HGF expression levels and overexpression of c-Met are often associated with dismal prognosis due to highly aggressive tumor behaviors and increased tumor metastasis [1,2,10,11]. Based on these characteristics, c-Met and HGF have been considered as ideal therapeutic targets in cancer-specific treatments; thus, numerous pre-clinical studies targeting the HGF/c-Met axis have been reported [12,13,14].

HGF/c-Met axis targeting therapeutic antibodies have been developed to prevent molecular interaction between c-Met and its ligand HGF or to inactivate c-Met via protein degradation or shedding (release from the cell surface) [13,14]. Rilotumumab (AMG102) and ficlatuzumab (AV-299) are HGF-neutralizing antibodies that only inhibit HGF-dependent c-Met activation by disrupting the HGF/c-Met interaction [15,16,17]. DN30 is a bivalent c-Met-targeted antibody that has been developed to deplete c-Met by shedding (proteolytic cleavage) the extracellular domain of c-Met, but an interaction between DN30 and c-Met results in the activation of the c-Met signaling pathway, inducing c-Met receptor homodimerization [18,19]. Bivalent c-Met-targeted antibodies impairing both HGF-dependent and independent activation rendered disappointing results, as their mechanisms could potentially induce agonistic effects via receptor dimerization [20]. Through extensive research and development to minimize the agonist activity of c-Met antibodies, onartuzumab (MetMab, formerly huOA5D5.v2), emibetuzumab (LY2875358), and SAIT301 were developed. Onartuzumab is an engineered monovalent c-Met antibody that was developed to prevent agonist activity with knob-into-hole technology, which allows the antibody to directly interact with c-Met in a one-on-one fashion [21,22]. Emibetuzumab and SAIT301 are humanized bivalent anti-c-Met monoclonal antibodies that were generated to prevent HGF from binding to c-Met and to induce antibody internalization, subsequently depleting c-Met receptors from the cell surface without inducing functional agonist activity [23,24]. However, clinical trials of onartuzumab were terminated after the drug failed to exhibit tumor inhibitory effect in NSCLC [25]. Other c-Met-targeting antibodies also failed to portray any favorable clinical outcomes.

Heterotopic or orthotopic tumor mouse models have been used extensively for the pre-clinical evaluation of human c-Met-specific therapeutic antibodies [15,16,17,18,19,20,21,22,23,24]. However, a precise assessment of the therapeutic efficacy of anti-human c-Met antibodies in mouse xenograft models could be hampered, as the reactivity of antibodies is restricted to the engrafted human tumor cells. Consistently, the abovementioned c-Met antibodies bind specifically to human c-Met, but do not engage with the mouse c-Met ortholog of mouse normal tissues [18,19,21,22,23,24], thus resulting in misleading interpretations in pre-clinical mouse models [26]. Previous reports have shown cross-reactive therapeutic antibodies to both the human protein, and mouse ortholog exhibited more accurate tumor inhibitory effects in mouse models, suggesting the necessity of developing cross-reactive antibodies [27,28,29].

In an effort to overcome this potential problem, we developed IRCR201—a novel human and mouse cross-reactive c-Met-targeting antibody. IRCR201 is a bivalent fully-human antibody that binds to the plexin-semaphorin-integrin (PSI) domain of c-Met and exhibits a tumor inhibitory effect by regulating tumor cellular growth, proving to have ideal effects in cancer treatment. IRCR201 also demonstrates low agonistic activity and disrupts c-Met signaling pathway activation induced by both HGF-dependent and independent mechanisms in various cancer types, eliciting distinct molecular traits compared to other c-Met-targeting antibodies. In addition, IRCR201 provides exceptional anti-tumor activity in both gastric cancer and NSCLC xenograft models. Collectively, our results demonstrate that targeting the PSI domain of c-Met provides a promising therapeutic approach, and its human and mouse cross-reactivity allows successful preclinical studies to assess precise therapeutic efficacy, pharmacokinetics (PK), and toxicity profiles, resulting in the further development of IRCR201 as an ideal cancer-targeted therapeutic agent.

## 2. Results

### 2.1. IRCR201 Exhibits High Affinity to Both Human and Mouse c-Met

**Figure 1:**
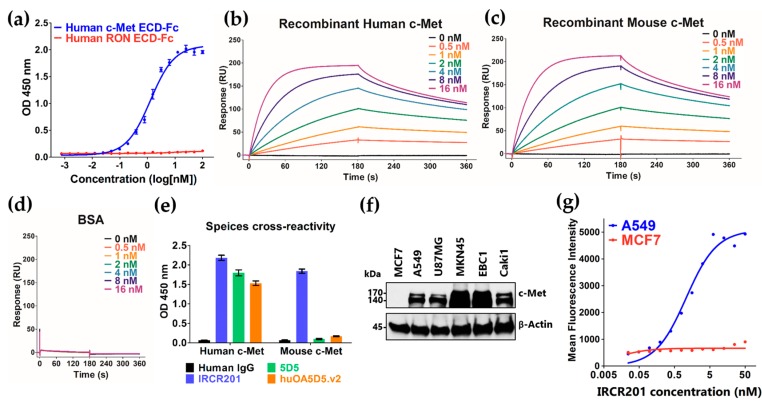
Binding properties of IRCR201. (**a**) Binding patterns of IRCR201 to the human c-Met extracellular domain (ECD)-fragment crystallizable region (Fc) and the human RON (recepteur d’origine nantais) ECD-Fc were measured by enzyme-linked immunosorbent assay (ELISA). IRCR201 binds to the human c-Met ECD-Fc with specificity and selectivity; (**b**–**d**) Surface plasmon resonance (SPR) sensorgrams binding with varying concentrations of IRCR201 to human c-Met, mouse c-Met, or bovine serum albumin (BSA) immobilized onto a CM5 Biacore^TM^ sensor chip; (**e**) Cross-reactivity analysis of IRCR201 to human and mouse c-Met; (**f**) Analysis of c-Met expression in various types of cancer cell lines; (**g**) Binding analysis of IRCR201 to the cell surface c-Met through a flow cytometer (FACSAria™ III).

IRCR201 is a cross-reactive c-Met-targeting human immunoglobulin G1 (IgG1), and was developed through panning techniques using synthetic human antibody phage libraries. To evaluate whether IRCR201 specifically binds to the c-Met extracellular domain (ECD)-fragment crystallizable region (Fc), we performed enzyme-linked immunosorbent assay (ELISA) using recombinant human c-Met and recepteur d’origine nantais (RON) proteins. The RON receptor tyrosine kinase is a member of the c-Met family, and has similar biochemical and structural properties to c-Met [30,31,32]. We confirmed that IRCR201 specifically binds to human c-Met in a dose-dependent manner, but not to the RON protein, indicating the selectivity of IRCR201 against the c-Met protein (Figure 1a). The binding kinetics of IRCR201 to recombinant human and mouse c-Met ECD was determined using Biacore™ T100 based on surface plasmon resonance (SPR). Our analysis showed that IRCR201 binds to human c-Met ECD-Fc with a K_D_ of 0.7207 nM, and mouse c-Met ECD-Fc with a K_D_ of 0.8448 nM (Figure 1b,c and Table 1). However, IRCR201 does not bind to bovine serum albumin (BSA) (Figure 1d). Additionally, IRCR201 demonstrates strong cross-reactivity to both human and mouse c-Met, representing differential binding properties compared to other c-Met-targeting antibodies such as 5D5 and huOA5D5.v2 (onartuzumab) (Figure 1b–e). To confirm the expression of c-Met in various cancer cell lines, we performed immunoblot analysis using commercial c-Met antibody (Figure 1f). To assess whether IRCR201 binds to the c-Met on the cell surface, the reactivity of IRCR201 against the cell surface c-Met of A549 (c-Met+) or MCF7 (c-Met−) was confirmed by flow cytometry. Overall, our results showed that IRCR201 specifically binds to the A549 cell surface in a dose-dependent manner, but does not engage to MCF7 lacking c-Met (Figure 1g).

### 2.2. IRCR201 Specifically Binds to the PSI Domain of c-Met

**Figure 2:**
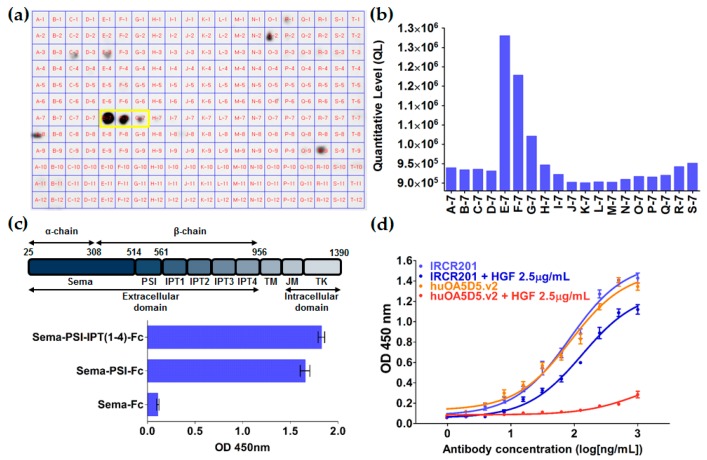
Epitope mapping of IRCR201. (**a**,**b**) The reactivity of IRCR201 against a panel of overlapping peptides representing the c-Met extracellular domain was determined by peptide array. A peptide library spanning amino acids 1–932 of the c-Met extracellular domain was synthesized (JPT Peptide Technologies GmbH, Berlin, Germany). The library was prepared as overlapping linear peptides covalently bound to a cellulose membrane. The results show dot blots of specific epitope sequences for IRCR201 (yellow frame). The quantified levels of dot intensity were determined by Multi Gauge V3.0 program; (**c**) Binding pattern analysis using domain proteins of c-Met; (**d**) Hepatocyte growth factor (HGF)/c-Met competitive ELISA. After the HGF (2.5 μg/mL) was pre-treated with the human c-Met protein-immobilized plates, IRCR201 or huOA5D5.v2 was added to confirm whether HGF and each antibody were competitively bound. IPT: immunoglobulin–plexin–transcription; PSI: plexin-semaphorin-integrin domain.

To determine the binding epitope of IRCR201 against c-Met, we performed epitope mapping using the IRCR201 single-chain variable fragment (scFv) format (Figure 2a, Table 2). The binding intensity of the epitope mapping analysis was quantified using Multi Gauge software V3.0, an image analysis program (Figure 2b, Table 2). Our results indicated that the scFv binds to E-7, F-7, and G-7 peptides spanning the Phe523–Cys545 amino acids, demonstrating that the scFv specifically recognizes an epitope within the PSI domain (Figure 2a,b and Table 2). Key residues of c-Met and scFv interaction were further validated through the SAPPFVQ (Ser531-Gln537) amino acid sequence (Figure 2a,b and Table 2). Although the binding activity is mediated by a small number of contacts, such types of interactions are extremely specific. Furthermore, we conducted binding pattern analysis using recombinant human c-Met domain proteins for additional verification. Our result showed that IRCR201 exclusively binds to the recombinant proteins with the PSI domain (Figure 2c). To confirm whether IRCR201 prevents molecular interaction between HGF and c-Met, competitive ELISA was conducted at fixed HGF concentration (2.5 µg/mL). The results showed that huOA5D5.v2 significantly inhibited the interaction between HGF and c-Met, whereas IRCR201 did not exhibit such behavior (Figure 2d).

### 2.3. IRCR201 Docks onto the PSI Domain of c-Met in Computational Modeling Analysis

**Figure 3:**
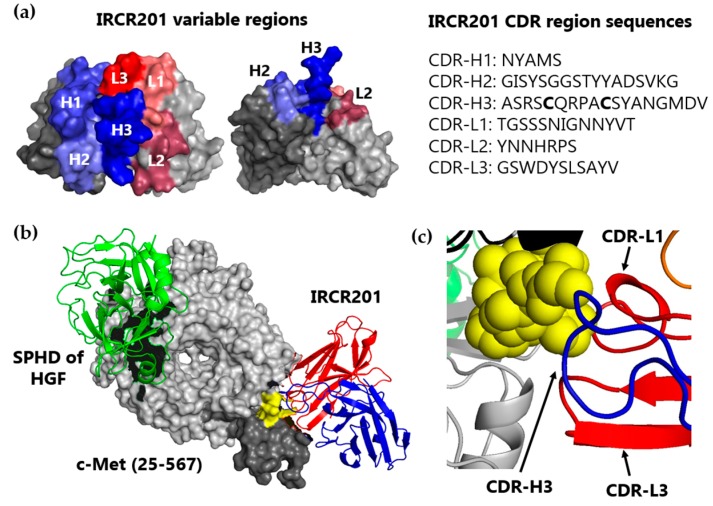
Docking of IRCR201 to human c-Met. (**a**) A three-dimensional (3D) model of IRCR201 variable domains was generated in a single-chain variable fragment (scFv) format by Rosetta computational homology modeling. The complementarity determining regions (CDRs) of the V_H_ and V_L_ domains are shown in blue and red, respectively. The framework regions of the V_H_ and V_L_ domains are represented in dark grey and light grey, respectively. The CDR sequences of IRCR201 are represented according to the Kabat numbering scheme; (**b**,**c**) IRCR201 was docked to c-Met (PDB accession: 1SHY) using the ZDOCK docking program. Epitope was mapped on to the 3D structure of c-Met (25–567), incorporating the Sema domain and plexin-semaphorin-integrin (PSI) domain. The figures were drawn with PyMOL (DeLano Scientific LLC, Palo Alto, CA, USA). The V_H_ and V_L_ domains of IRCR201 are shown in blue and red, respectively. Yellow = IRCR201 binding site (SAPPFVQ). Light grey = Sema domain. Dark grey = PSI domain. Green = serine protease homology domain (SPHD) of HGF.

To expand the structural understanding of the docking of IRCR201 onto c-Met, computational modeling was conducted using a published c-Met structure (PDB accession: 1SHY) [33]. A three-dimensional (3D) structural model of IRCR201 scFv in the docking region of the c-Met structure was generated using the Rosetta-based computational homology modeling technique [34] (Figure 3a). Our results showed that IRCR201 possesses an elongated complementarity-determining region (CDR)-H3 consisting of 18 amino acids, and the extended CDR-H3 loop is stabilized through interchain disulfide bond between the cysteines of CDR-H3 (Figure 3a). We used ZDOCK—a protein-protein docking program—to aid in the structural understanding of the interaction between c-Met and IRCR201 and to observe whether IRCR201 scFv docks onto the specific epitope (yellow) within the PSI domain (dark gray) [35] (Figure 3b). The binding site of IRCR201 was on the PSI domain (dark gray), whereas the serine proteinase homology domain (SPHD) of HGF binds to the Sema domain (light gray). The distance between the two binding sites was considerably remote, proving that IRCR201 does not interfere with the interaction between HGF and c-Met in the 3D structure analysis. In this docking model, the CDR-H3, CDR-L1, and CDR-L3 of IRCR201 showed strong interaction with the epitope (Figure 3c).

### 2.4. IRCR201 Exhibits Low Agonistic Activity

**Figure 4:**
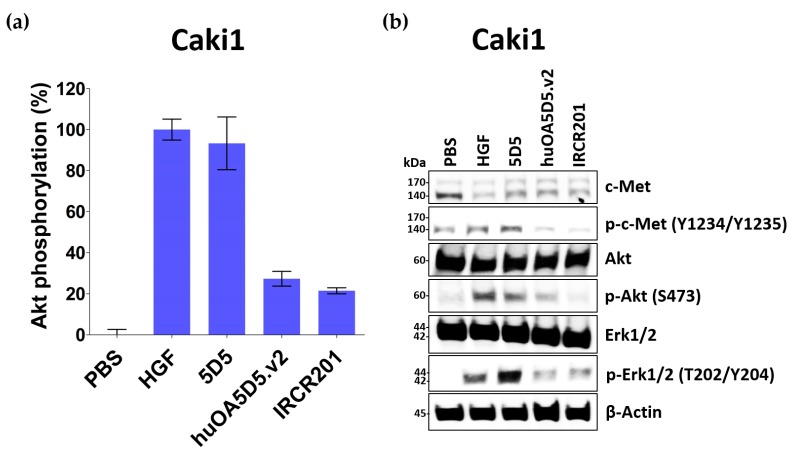
Agonist activity assay. Caki1 cells were starved in serum-free medium for 24 h and incubated with 100 nM of c-Met antibodies or 50 ng/mL of HGF for 30 min. (**a**) Akt phosphorylation in Caki1 cells was measured by ELISA; (**b**) Western blot analysis of c-Met, phospho-c-Met, Akt, phospho-Akt, Erk1/2, phospho-Erk1/2, and β-actin.

In the development of c-Met inhibitory antibodies, the bivalent nature of the c-Met antibodies enabled the antibodies to mimic the role of HGF and thus activate the c-Met signaling pathway [18]. Because Akt acts as the main mediator of the c-Met signaling pathway [3], we investigated Akt signaling activity to evaluate the agonist activity of c-Met antibodies in the Caki1 renal cell carcinoma cell line (Figure 4a). In our assay, IRCR201 was compared with HGF and 5D5 as positive controls. As a result, we confirmed that 5D5—a c-Met agonistic antibody—phosphorylates Akt to a similar extent as HGF. huOA5D5.v2 is a monovalent antibody engineered to minimize agonistic activity, and showed Akt phosphorylation levels at 27.3% compared to the phosphate-buffered saline (PBS)-treated group, and IRCR201 exhibited a similar Akt phosphorylation level at 21.5% (Figure 4a). In addition, we further confirmed IRCR201-triggered agonistic effect by investigating the HGF/c-Met signaling pathway in Caki1 renal cell carcinoma cell line (Figure 4b). As a result, IRCR201 exhibited low agonist activity compared to HGF or 5D5 (Figure 4b). Although IRCR201 is a bivalent antibody, it demonstrated a lower agonistic effect compared to the modified monovalent antibody with minimal agonist activity.

### 2.5. IRCR201 Impedes Tumor Growth and Induces Cellular Apoptosis

**Figure 5:**
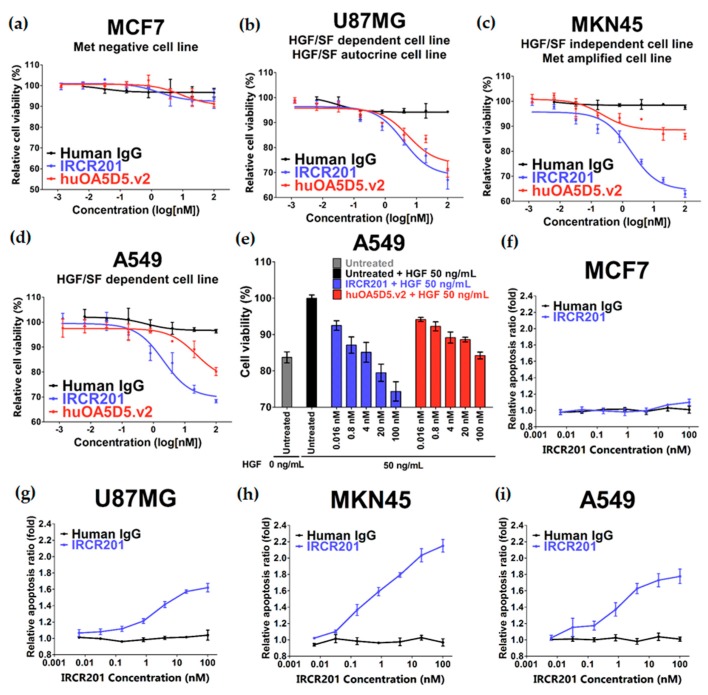
In vitro potency of IRCR201. All results are shown as the mean ± standard error of mean (SEM) from triplicate treatments. (**a**–**d**) Inhibitory effect of IRCR201 on cancer cell proliferation. MCF7, U87MG, MKN45, and A549 cells were treated with IRCR201, huOA5D5.v2, or human IgG control for 72 h. Cell proliferation was measured using CellTiter Glo^®^ (Promega); (**e**) HGF-induced growth inhibition by IRCR201. The inhibitory effect of IRCR201 and huOA5D5.v2 on cell growth was examined under the condition of the addition of 50 ng/mL HGF in A549, an HGF-dependent cell. After 72 h of antibody treatment, the number of cells was measured with CellTiter Glo^®^ (Promega); (**f**–**i**) Apoptosis assay. MCF7, U87MG, MKN45, and A549 cells were treated with IRCR201 or human IgG for 24 h. Apoptosis activity was detected with caspase-3/7 activity.

In vitro functional inhibition activity of IRCR201 was analyzed in various cancer types. IRCR201 did not alter cancer cell growth of the low c-Met-expressing cell line MCF7 (Figure 1f and Figure 5a). In U87MG (an HGF-dependent GBM cell line), IRCR201 showed more potent cancer cell growth inhibition compared to huOA5D5.v2, which has previously been reported to suppress only HGF-dependent cellular proliferation (Figure 5b) [19,20]. To determine whether IRCR201 could inhibit the tumor growth of cancer cells with c-Met amplification and constitutively phosphorylate c-Met activity in the absence of HGF, an MKN45 gastric cancer cell line was employed and showed significantly reduced IRCR201-mediated cellular proliferation compared to huOA5D5.v2 (Figure 5c). Additionally, we used an A549 lung cancer cell line, which has been previously demonstrated to secrete no detectable level of HGF but could potentially promote c-Met activation in response to HGF, for further assessment of cellular proliferation in the presence of IRCR201. In the absence of HGF, IRCR201 displayed more potent cancer cell growth inhibition in A549 compared to huOA5D5.v2 (Figure 5d). Furthermore, the inhibitory effect of HGF-induced cancer cell growth of IRCR201 was superior to that of huOA5D5.v2 (Figure 5e). We also confirmed that IRCR201 could dramatically induce cellular apoptosis in c-Met-expressing cancer cell lines except for MCF7 (c-Met expression: low) (Figure 5f–i).

### 2.6. IRCR201 Induces Rapid c-Met Internalization and Lysosomal Degradation

**Figure 6:**
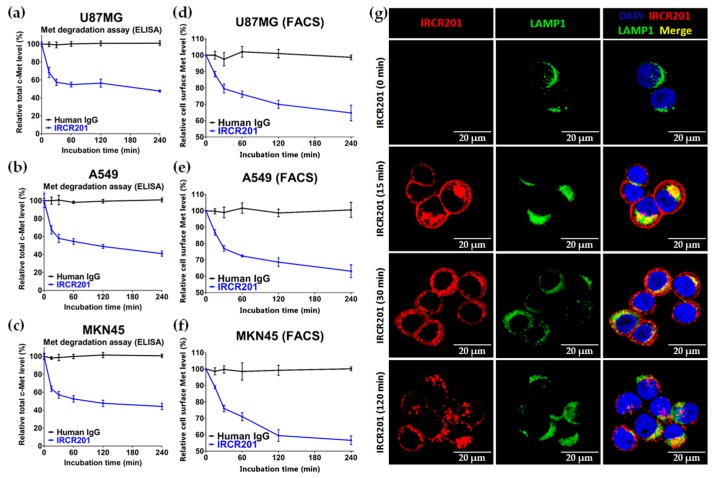
Effect of IRCR201 on c-Met receptor internalization and degradation. (**a**–**c**) U87MG, A549, and MKN45 were treated with 100 nM IRCR201 or human IgG for the indicated durations and lysed. The level of total c-Met was measured by ELISA-based assay as described in the “Materials and Methods” section; (**d**–**f**) After c-Met-positive cell lines were incubated with 100 nM of IRCR201 or human IgG for 30 min at 4 °C, the cell lines were incubated at 37 °C to induce internalization and degradation of the c-Met/IRCR201 complex. The residual levels of cell surface c-Met were detected by rabbit anti-c-Met antibody-conjugated Alexa Fluor^®^ 488 in which the epitope of IRCR201 does not overlap. The c-Met levels on the cell surface were measured based on the mean fluorescence intensity (MFI) and normalized to a value of MFI at 0 min; (**g**) MKN45 cells were treated with 100 nM IRCR201 at 37 °C. Thereafter, IRCR201 was allowed to internalize for up to 120 min at 37 °C. MKN45 cells were then stained with anti-human IgG-Alexa Fluor^®^ 647 to recognize IRCR201 and anti-LAMP1-Alexa Fluor^®^ 488 to detect lysosomes. The fluorescence images are visualized by confocal microscopy.

**Figure 7:**
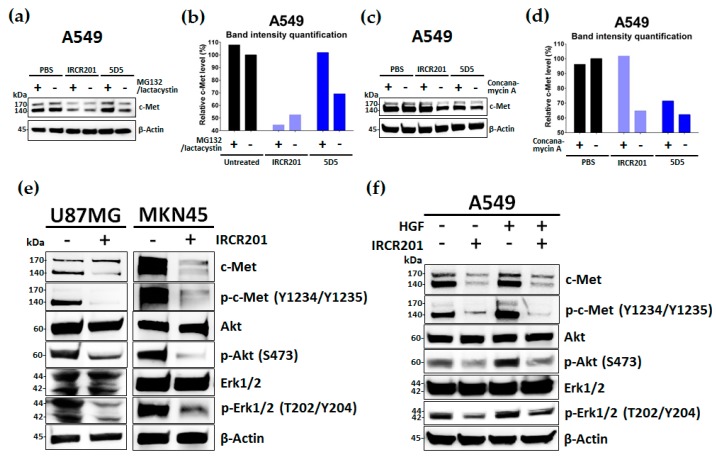
IRCR201 suppresses c-Met signaling pathway via the degradation of c-Met. (**a**,**b**) A549 cells were pre-treated with dimethyl sulfoxide (DMSO) or MG132 (5 μM)/lactacystin (5 μM) mixture for 2 h and incubated with 100 nM anti-c-Met antibodies (IRCR201 or 5D5) or phosphate-buffered saline (PBS). After 4 h of incubation, the c-Met degradation pattern was measured by Western blot analysis. The band intensities of c-Met were quantified by a Multi Gauge V3.0 program and normalized by the intensity of corresponding β-actin band; (**c**,**d**) A549 cells were pre-treated with DMSO or 100 nM concanamycin A for 2 h and subsequently treated with 100 nM anti-c-Met antibodies (IRCR201 or 5D5) or PBS for 4 h. The c-Met degradation pattern was evaluated by immunoblot analysis and analyzed by Multi Gauge V3.0 program; (**e**) Western blot analysis of c-Met, phospho-c-Met, Akt, phospho-Akt, Erk1/2, and phospho-Erk1/2 after 24-h treatment with IRCR201 (100 nM) or PBS in U87MG and MKN45; (**f**) Western blot analysis of c-Met, phospho-c-Met, Akt, phospho-Akt, Erk1/2, and phospho-Erk1/2 after 24-h treatment with IRCR201 (100 nM) or PBS in A549 in the presence or absence of HGF (50 ng/mL).

To elucidate the cellular growth inhibitory mechanism of IRCR201 in c-Met-expressing cancer cell lines, we measured the change in total c-Met protein levels according to antibody treatment time in various cancer cell lines using ELISA. In multiple cancer cell lines, the total c-Met protein level was rapidly attenuated after 15 min from the initial treatment with IRCR201 (Figure 6a–c). Four hours after the IRCR201 treatment, the total c-Met level was reduced to 52.4, 60.0, and 56.8% in U87MG, A549, and MKN45, respectively (Figure 6a–c). We further analyzed the molecular interaction between IRCR201 and c-Met receptors on the cell surface in various c-Met-overexpressed cancer cell lines. Our results highlighted the decomposition of cell surface c-Met via IRCR201 treatment (Figure 6d–f). The resulting surface c-Met levels 4 h post-treatment were 35.4%, 37.0%, and 44.5% in U87MG, A549, and MKN45, respectively. Immunocytochemistry analysis was performed on MKN45 to clarify whether the reduced level of c-Met on the cell surface was due to the degradation of antibody-mediated internalization. The results showed that IRCR201 had progressed into the cytoplasmic area of MKN45, eventually exhibiting co-localization with lysosomal-associated membrane protein 1 (LAMP1, a lysosomal marker), inducing the efficient degradation of cell surface c-Met (Figure 6g). To investigate the underlying mechanism behind the IRCR201-mediated c-Met degradation, we analyzed the c-Met degradation patterns of IRCR201 or 5D5 in the presence of a proteasome inhibitor or lysosome inhibitor. Numerous studies have reported that the binding of HGF or 5D5 with c-Met induces internalization of the ligand/receptor complex and initiates the degradation of receptor tyrosine kinase (RTK) through the ubiquitin–proteasome pathway [24,36,37,38]. IRCR201 dramatically decomposed c-Met in A549 under specific conditions where MG132 and lactacystin (proteasome inhibitors) were first treated, but 5D5 failed to degrade c-Met (Figure 7a,b). Treatment with concanamycin A (a lysosome inhibitor) failed to induce IRCR201-mediated c-Met degradation in A549, confirming that IRCR201-induced c-Met depletion is mediated by the lysosomal degradation pathway (Figure 7c,d). Our results further showed that the 5D5 antibody could induce c-Met degradation, whether in the presence or absence of concanamycin A (Figure 7c,d). In conclusion, these results indicate that IRCR201 mediates endogenous c-Met depletion through the lysosomal degradation pathway.

### 2.7. IRCR201 Suppresses the c-Met Signaling Pathway via the Degradation of c-Met

Immunoblot analysis of HGF-dependent (U87MG) and HGF-independent c-Met-amplified (MKN45) cell lines were conducted to analyze the inhibitory effect of IRCR201 on the c-Met signaling pathway. In both models, IRCR201 efficiently depleted total c-Met levels, and inhibited c-Met phosphorylation and its downstream signaling pathway (Figure 7e). In addition, we further confirmed the inhibition of the c-Met pathway by IRCR201 under the presence of HGF in A549, an HGF-dependent lung cancer cell line. In the absence of HGF, IRCR201 exhibited exceptional c-Met degradation, subsequently inhibiting phosphorylation of c-Met downstream pathway molecules including Akt and Erk1/2 (Figure 7f). In the presence of HGF, IRCR201 treatment not only promoted c-Met degradation, but also inhibited the phosphorylation of c-Met, Akt, and Erk1/2 (Figure 7f). Overall, our results demonstrated that IRCR201 can efficiently interfere with the HGF/c-Met axis, regardless of HGF-dependent or -independent growth mechanisms.

### 2.8. IRCR201 Impedes Tumor Growth In Vivo

**Figure 8:**
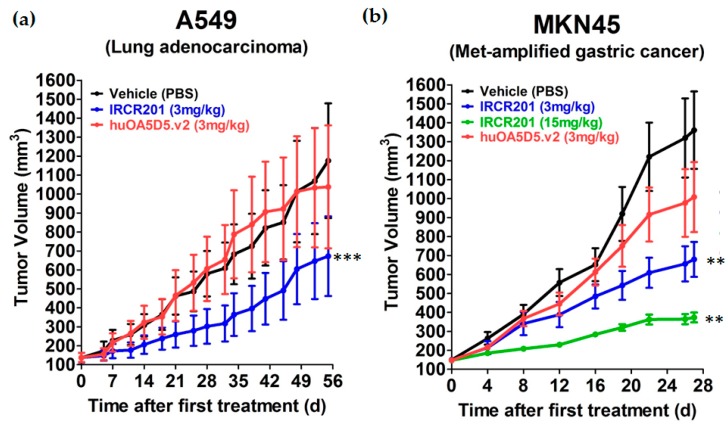
In vivo potency of IRCR201 in c-Met-expressing tumor xenograft models. (**a**) IRCR201 was dosed intravenously twice a week at 3 mg/kg in an A549 non-small cell lung cancer (NSCLC) xenograft model, as compared with the PBS-treated group or huOA5D5.v2-treated group; (**b**) IRCR201 was intravenously injected at 3 or 15 mg/kg twice a week in an MKN45 gastric cancer xenograft model compared with the PBS-treated group or huOA5D5.v2-treated group. Six mice per group were used for the A549 and MKN45 studies. Asterisks (*) indicate *p*-values versus PBS-treated group (vehicle group) according to one-tailed two-sample *t*-test. *p*-Value < 0.05 was considered statistically significant. (** *p* < 0.01, *** *p* < 0.001).

In vivo anti-tumor activity of IRCR201 was observed in an A549 NSCLC subcutaneous model. Our results showed that 3 mg/kg IRCR201 treatment (48.4% tumor inhibition) could potently inhibit tumor growth, while 3 mg/kg huOA5D5.v2 treatment (13.3% tumor inhibition) showed a similar tumor growth rate compared to the PBS-treated group (Figure 8a). In addition, we further analyzed whether IRCR201 could significantly inhibit c-Met-amplified tumor models. The c-Met-amplified gastric cancer cell line MKN45 was subcutaneously inoculated into BALB/c-nu mice. IRCR201 was injected intravenously twice a week at 3 or 15 mg/kg, and significant inhibition in tumor growth was observed compared to the PBS-treated group (Figure 8b). The 3 mg/kg IRCR201-treated group (56.3% tumor inhibition) showed significant reduction in tumor volume compared to the 3 mg/kg huOA5D5.v2-treated group (29.1% tumor inhibition) (Figure 8b). When we increased the IRCR201 dose to 15 mg/kg (81.5% tumor inhibition), the tumor growth rate was significantly diminished compared to the 3 mg/kg IRCR201-treated group, portraying a dose-dependent reduction of tumor volume compared to the vehicle-treated group (Figure 8b).

### 2.9. IRCR201 Inhibits Tumor Cell Proliferation and Angiogenesis through the Downregulation of c-Met

**Figure 9:**
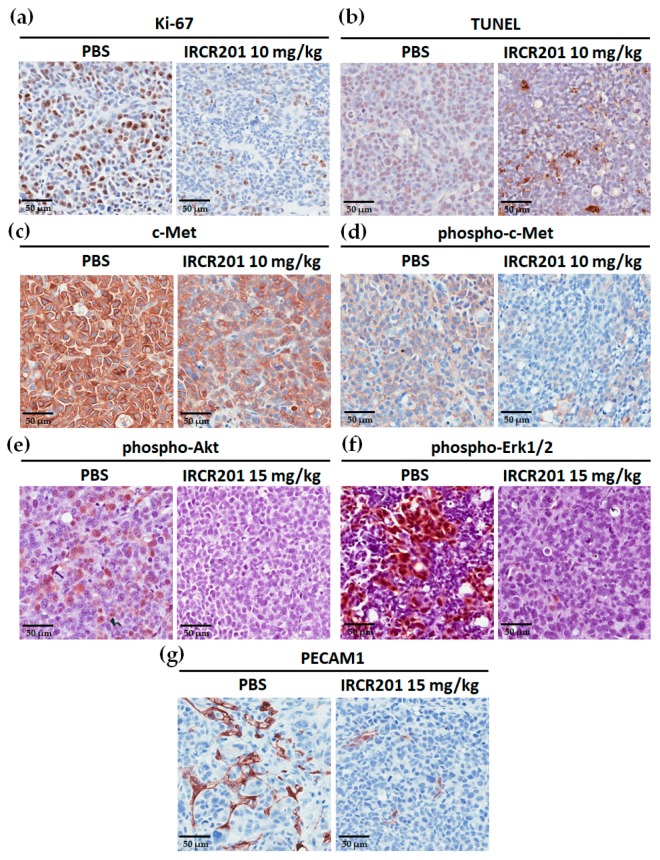
Immunohistological staining of Ki-67, apoptotic cells, c-Met, phospho-c-Met, phospho-Akt, phospho-Erk1/2, and platelet endothelial cell adhesion molecule 1 (PECAM1) in an MKN45 xenograft mouse tumor model. (**a**–**g**) All the immunohistochemistry (IHC) images were obtained at the same magnification. Sections were counterstained with hematoxylin (blue); (**a**–**d**) Immunohistochemistry analysis of MKN45 tumors in mice at 48 h post-treatment with 10 mg/kg IRCR201. The control group was intravenously injected with PBS (vehicle); (**a**) Immunohistological staining of MKN45 tumor section for Ki-67 (brown); (**b**) Immunohistochemistry analysis of apoptotic cells (brown) using the terminal deoxynucleotidyl transferase dUTP nick end labeling (TUNEL) assay; (**c**,**d**) IHC expression images of total c-Met (brown) and phospho-c-Met (brown) in the MKN45 tumor section at 48-h post-treatment at 10 mg/kg; (**e**–**g**) Immunohistochemistry analysis of phospho-Akt, phospho-Erk1/2, and PECAM1 on 15 mg/kg IRCR201-treated MKN45 tissue sections; (**e**,**f**) IHC expression images of phospho-Akt (brown) and phospho-Erk1/2 (brown) in the MKN45 tumor sections; (**g**) Immunohistological analysis of PECAM1-positive blood vessels (brown) in the 15 mg/kg IRCR201-treated MKN45 xenograft tumor section.

Based on the inhibitory effects of IRCR201 in vitro and in vivo, the cellular mitotic index was further evaluated through immunohistochemical analysis of Ki-67-positive nuclei in the paraffin sections of MKN45 xenograft models that had been treated with either PBS (vehicle) or 10 mg/kg IRCR201 and harvested after 48 h. Immunohistochemistry (IHC) staining showed that Ki-67 positive cells were significantly reduced in the IRCR201-treated group compared to the PBS-treated group (Figure 9a). Additionally, immunohistochemical analysis in MKN45 tumor tissues demonstrated a significant increase of IRCR201-triggered terminal deoxynucleotidyl transferase dUTP nick end labeling (TUNEL)-positive apoptotic cells (Figure 9b). To investigate whether IRCR201 could induce c-Met degradation and phospho-c-Met inhibition in vivo, both total c-Met and phospho-c-Met levels were evaluated. Our results showed reduced expression of both total c-Met and phospho-c-Met when administered with IRCR201 at 10 mg/kg (Figure 9c,d). To investigate the status of downstream mediators in HGF/c-Met signaling pathway by IRCR201 treatment, we evaluated phosphorylation of Akt and Erk1/2 in PBS- or 15 mg/kg IRCR201-treated MKN45 tumor sections through immunohistochemical analysis (Figure 9e,f). The data demonstrated that IRCR201 significantly abrogated the phosphorylation of Akt and Erk1/2 in vivo (Figure 9e,f). To assess the anti-angiogenic effects of IRCR201, paraffin sections of MKN45 tumors were immuno-stained with platelet endothelial cell adhesion molecule 1 (PECAM1, also known as CD31), an endothelial cell marker (Figure 9g). The number of PECAM1-positive cells was drastically reduced in MKN45 tumors treated with 15 mg/kg IRCR201 compared to the PBS-treated group (Figure 9g).

## 3. Discussion

Aberrant activation of the HGF/c-Met signaling pathway has been reported in numerous human cancers [1,2]. In c-Met dysregulated malignancy, tumor progression is facilitated largely by three mechanisms: ligand-dependent c-Met activation; genomic amplification; and oncogenic mutations [5,6,7,8,9]. Additionally, c-Met amplification has been associated with acquired resistance to epidermal growth factor receptor (EGFR)- and vascular endothelial growth factor (VEGF)-targeted therapies [39,40]. Therefore, targeting the HGF/c-Met signaling axis could offer a promising therapeutic approach for the treatment of c-Met-expressing tumors. In the present study, we demonstrated the development and characterization of IRCR201—novel fully-human anti-c-Met IgG1 which specifically binds to a distinct epitope on c-Met and effectively disrupts the c-Met signaling pathway. In contrast to previously developed c-Met-targeting antibodies with lack of mouse cross-reactivity, IRCR201 binds to both human and mouse c-Met with high affinity. Cross-reactivity with the mouse c-Met ortholog enables the precise evaluation of the tumor inhibitory efficacy of IRCR201 in mouse xenograft models during preclinical studies.

IRCR201 does not mimic the role of HGF and exhibits lower agonist activity compared to huOA5D5.v2 as it binds to the SAPPFVQ amino acid sequence of the PSI domain, as distinct from the Sema domain, which is an HGF binding site of c-Met. The PSI domain containing the SAPPFVQ sequence—the IRCR201 epitope—acts as a domain that gives flexibility to c-Met, promoting molecular interaction between c-Met and HGF [41,42]. Although the biological insights of the PSI domain remain relatively unexplored, IRCR201 interacts with the PSI domain of c-Met, thus resulting in an inhibitory effect without agonist activity. Basilico and colleagues also developed specific antibodies that bind to the PSI domain and disrupt the interaction between HGF and c-Met [43], resulting in the inhibition of HGF-induced c-Met autophosphorylation, distinct from IRCR201, which does not impair the interaction between c-Met and HGF. Previous studies have reported that bivalent c-Met-targeting antibodies with different epitopes could elicit different levels of biochemical functional activity [18,43,44]. The differential epitope of c-Met antibodies may account for the distinct functional activity of IRCR201.

IRCR201 possesses a different binding epitope from previously developed c-Met inhibitory antibodies, and rapidly promoted c-Met depletion primarily through the lysosomal degradation pathway, thereby abrogating downstream signaling cascades in U87MG, A549, and MKN45. To further ascertain the effect of IRCR201 at a cell surface receptor level, a flow cytometry assay was configured to detect cell surface c-Met of U87MG, A549, and MKN45 following the antibody treatment. Similar results were observed throughout various cell lines, as the rapid degradation of cell surface c-Met was observed at the 15-min mark. Total receptor expression levels in the membrane were decreased by approximately 40% post IRCR201 treatment. Furthermore, we found that IRCR201 resulted in c-Met co-localization within the lysosome, suggesting an underlying mechanism behind IRCR201-induced c-Met degradation. The c-Met depletion activity of IRCR201 was inhibited by concanamycin A, supporting the notion that IRCR201 deteriorates c-Met through the lysosomal degradation pathway. These findings demonstrate that IRCR201 induces receptor-mediated internalization and lysosomal degradation, providing a potential underlying mechanism behind the loss of total c-Met protein.

IRCR201 does not inhibit the interaction between HGF and c-Met, but it exhibited excellent growth inhibition capacity in various types of c-Met-expressing cancer cell lines compared to huOA5D5.v2 in vitro. IRCR201-induced c-Met degradation demonstrated a therapeutic effect mediated by the downregulation of tyrosine kinase activity and the subsequent inhibition of cellular proliferation in U87MG, A549, and MKN45. IRCR201 also showed significant antitumor effect in an A549 NSCLC xenograft tumor model. Since c-Met amplification leads to shorter survival in patients with gastric cancer and NSCLC [45,46], we assessed the tumor inhibitory capability of IRCR201 in a c-Met-amplified tumor model to provide clinical benefit in patients. Treatment with IRCR201 dramatically decreased tumor growth in the c-Met-amplified MKN45 gastric xenograft model, suggesting that it may portray antitumor activity in a broader range of cancer classes with constitutive c-Met activation via genomic amplification or mutations. These results suggest that IRCR201 could be a promising therapeutic agent to inhibit tumor growth driven by constitutively active c-Met through overexpression, gene amplification, or genomic mutations.

The HGF/c-Met signaling pathway is a pivotal component of tumor-associated angiogenesis that leads to tumor progression and metastasis via a sufficient supply of oxygen and nutrients through blood vessels [1,2]. Immunohistochemistry analysis also revealed that IRCR201 significantly inhibited the proliferation of tumor cells and angiogenesis of tumor-associated blood vessels, suggesting that IRCR201 suppresses the HGF/c-Met pathway in the tumor microenvironment.

Previous efforts to develop bivalent c-Met antagonistic antibodies were unsuccessful, as the antibodies incited agonistic effects [20]. Through the comprehensive research of c-Met biology, numerous research groups have developed various c-Met antibodies such as onartuzumab (huOA5D5.v2), emibetuzumab (LY2875358), telisotuzumab (ABT-700), SAIT301, ARGX-111, and DN30 [21,22,23,24,43,44,47,48], which have diverse molecular mechanisms of action in downregulating the functional activity of c-Met. Among these, emibetuzumab and SAIT301—the c-Met inhibitory antibodies—have similar c-Met degradation activity to that of IRCR201; however, they have completely different binding epitopes from IRCR201.

By impairing both HGF-dependent and c-Met-amplified downstream activation in various cancer types, IRCR201 is differentiated from other therapeutic antibodies by securing a wide range of drug response groups. The anti-cancer activity of IRCR201 may be further enhanced by antibody-mediated cell cytotoxicity (ADCC), as the antibody’s isotype is human IgG1, which binds to the Fc receptors on immune cells. IRCR201 is also applicable to antibody–drug conjugate (ADC) platforms for toxin delivery, as it demonstrated the lysosomal degrading traits of c-Met.

This study currently has an accompanying research program to investigate the specific degradation pathway responsible for IRCR201-induced c-Met depletion. IRCR201 is also being used in toxicological studies using mouse models to confirm that its mouse cross-reactivity has an adverse effect on mouse health.

In summary, the newly identified traits of IRCR201 allow the evaluation of precise therapeutic efficacy in mouse models. Our comprehensive results show that IRCR201 is capable of inhibiting a variety of cancer types with HGF-dependent c-Met activation or gene amplification-driven constitutive c-Met activation. Taken together, IRCR201 represents a promising therapeutic antibody to treat cancer patients suffering from dysregulation of the HGF/c-Met signaling pathway.

## 4. Materials and Methods

### 4.1. Generation of Antibodies

Biopanning was performed using phage displayed synthetic human scFv libraries according to basic panning protocol [49]. Briefly, scFv displaying phage libraries were enriched on 1 µg immobilized human c-Met ECD-Fc (Sino Biological, 10692-H03H, Beijing, China) and mouse c-Met ECD-Fc (Sino Biological, 50622-M02H, Beijing, China) in MaxiSorp™ immune-tubes (Nunc, 444202, Roskilde, Denmark). c-Met specific binders were screened and selected by ELISA using produced scFvs in human and mouse c-Met-coated 96-well enzyme immunoassay/radioimmunoassay (EIA/RIA) plates (Costar, #3590, Corning, NY, USA). Finally, the selected scFv was reformatted to human IgG1, and named IRCR201. Antibody expression vectors of onartuzumab (huOA5D5.v2) were made using the amino acid sequences shown in patent US7892550 and in the published literature [22]. IRCR201 and huOA5D5.v2 were produced in an Expi293™ transient mammalian expression system (Gibco, A14635, Carlsbad, CA, USA) and purified by HiTrap™ Mabselect SuRe (GE Healthcare Life Sciences, 11-0034-93, Uppsala, Sweden). The anti-human c-Met agonistic antibody 5D5 was purchased in the form of hybridoma (American Type Culture Collection (ATCC), HB-11895, Manassas, VA, USA) and purified from hybridoma supernatant by HiTrap™ Protein G HP (GE Healthcare Life Sciences, 17-0405-01, Uppsala, Sweden). Control human IgG was purchased from Sigma (Sigma, I2511, St. Louis, MO, USA).

### 4.2. Cells and Cell Cultures

The MCF7 (ATCC, HTB-22), U87MG (ATCC, HTB-14), A549 (ATCC, CCL-185), and Caki-1 (ATCC, HTB-46) cell lines were obtained from American Type Culture Collection (ATCC, Manassas, VA, USA), and the MKN45 (JCRB Cell Bank, JCRB0254) cell line was purchased from the Japanese Collection of Research Bioresources Cell Bank (JCRB Cell Bank, Osaka, Japan). All cell lines were tested for mycoplasma, cross-contamination, and genetic fingerprints. U87MG were cultured in Eagle’s Minimum Essential Medium (EMEM; ATCC, 30-2003, Manassas, VA, USA) containing 10% (*v*/*v*) fetal bovine serum (FBS; Gibco, 26140079, Carlsbad, CA, USA) and 1% (*v*/*v*) penicillin/streptomycin (Gibco, 15140122, Carlsbad, CA, USA). MCF7, MKN45, A549, and Caki1 were cultured in Roswell Park Memorial Institute (RPMI) 1640 medium (Gibco, 11875093, Carlsbad, CA, USA) containing 10% (*v*/*v*) FBS (Gibco, 26140079, Carlsbad, CA, USA) and 1% (*v*/*v*) penicillin/streptomycin (Gibco, 15140122, Carlsbad, CA, USA). All cells were cultured according to the manufacturer's manual and had been passaged for fewer than three months after thawing. Cell counting was performed with the Scepter™ 2.0 automated cell counter (Millipore, Billerica, MA, USA) and c-Met expression was confirmed by Western blot using cell lysates.

### 4.3. Animals

BALB/c-nude mice (female) were used for the in vivo studies. All animals were obtained from Orient Bio Inc. (Seongnam, Korea), and were maintained under specific pathogen-free conditions in facilities approved by the Association for Assessment and Accreditation of Laboratory Animal (AAALAC) International in accordance with the current regulations and guidelines of the Laboratory Animal Research Center (LARC) at the Samsung Medical Center (Project identification code: NTX1161851-20160324002). Mice were acclimated for at least a week before they entered the studies.

### 4.4. Enzyme-Linked Immunosorbent Assay (ELISA)

To analyze the specificity and selectivity of IRCR201, 0.1 µg human c-Met protein (Sino Biological, 10692-H03H, Beijing, China) or 0.1 µg human RON protein (Sino Biological, 11608-H08H, Beijing, China) was coated in 96-well EIA/RIA plates (Costar, #3590, Corning, NY, USA) at 4 °C overnight. Following three washes with 1× phosphate-buffered saline with Tween 20 (PBST; Cell Signaling Technology, #9809, Danvers, MA, USA), they were blocked with 3% (*w*/*v*) skim milk (BD Difco, 232100, Sparks, MD, USA) for 1 h at room temperature. The wells of the plates were washed three times with 1× PBST and then incubated with 100 µL of IRCR201 diluted to the appropriate concentration for 1 h at room temperature. After three washes with 1× PBST, the wells of the plates were incubated with 100 µL of 1:10,000 goat anti-human IgG F(ab’)2 conjugated horseradish peroxidase (HRP) (Thermo Scientific, 31482, Waltham, MA, USA) for 1 h at room temperature, and then again washed three times with 1× PBST. 3,3′,5,5′-Tetramethylbenzidine (TMB) substrate solution (Thermo Scientific, N301, Waltham, MA, USA) was added to each well, and then reactions were quenched by STOP solution (Cell Signaling Technology, #7002, Danvers, MA, USA).

To investigate the species cross-reactivity of c-Met antibodies to human and mouse c-Met, 0.1 µg/well human c-Met ECD-Fc (Sino Biological, 10692-H03H, Beijing, China) or 0.1 µg/well mouse c-Met ECD-Fc (Sino Biological, 50622-M02H, Beijing, China) was coated in 96-well EIA/RIA plates (Costar, #3590, Corning, NY, USA). The subsequent steps were performed as described above. Since 5D5 was a mouse antibody, goat anti-mouse Fab conjugated HRP (Abcam, ab6823, Cambridge, UK) was used as a secondary antibody.

To confirm the binding property of IRCR201 to human c-Met domain proteins, 96-well EIA/RIA plates (Costar, #3590, Corning, NY, USA) were immobilized with 100 µL/well of 0.1 µg recombinant human Sema-Fc (protein consists of amino acids 1 to 515 of human c-Met), 0.1 µg recombinant human Sema-PSI-Fc (protein consists of amino acids 1 to 562 of human c-Met), and 0.1 µg recombinant human whole c-Met ECD-Fc (Sino Biological, 10692-H03H, Beijing, China). Recombinant human c-Met domain proteins with an Fc tag (rhSema-Fc and rhSema-PSI-Fc) were expressed in an Expi293™ system (Gibco, Carlsbad, CA, USA) and purified from HiTrap™ Mabselect SuRe (GE Healthcare Life Sciences, 11-0034-93, Uppsala, Sweden). Then, 100 µL of IRCR201 (1 µg/mL) was used as the primary antibody, and 100 µL of 1:10,000 goat anti-human IgG F(ab’)2 conjugated HRP (Thermo scientific, 31482, Waltham, MA, USA) was used as the secondary antibody. The color of the plates was developed using 3,3′,5,5′-tetramethylbenzidine (TMB) substrate solution (Thermo scientific, N301, Waltham, MA, USA) and the reaction was terminated with STOP solution (Cell Signaling Technology, #7002, Danvers, MA, USA).

### 4.5. HGF Ligand-Blocking ELISA

For the HGF/c-Met blocking assay (competitive ELISA), 96-well EIA/RIA plates (Costar, #3590, Corning, NY, USA) were coated with recombinant human c-Met (1 μg/μL, 100 μL) at 4 °C overnight. After washing the wells with 1× PBST, they were blocked with 3% skim milk for 1 h at room temperature. After pre-binding with/without 2.5 μg/mL HGF (R&D Systems, 294-HG, Minneapolis, MN, USA) at room temperature for 1 h, IRCR201 and huOA5D5.v2 were incubated at room temperature for 1 h. After three washes with 1× PBST, the wells were incubated with 100 μL of 1:10,000 goat anti-human IgG F(ab’)2 conjugated HRP (Thermo scientific, 31482, Waltham, MA, USA) for 1 h and then washed three times with 1× PBST. TMB substrate solution (Thermo Scientific, N301, Waltham, MA, USA) was added to each well and incubated at room temperature until color was developed. Afterwards, reactions were quenched by STOP solution (Cell Signaling Technology, #7002, Danvers, MA, USA). Optical density (OD) of the wells was measured at 450 nm with an Infinite^®^ M200 pro (Tecan, Männedorf, Switzerland).

### 4.6. Surface Plasmon Resonance Assay

The binding affinities and kinetics of IRCR201 for human c-Met and mouse c-Met were measured using a Biacore™ T100 (GE Healthcare Life Sciences, Uppsala, Sweden). Human c-Met, mouse c-Met, or BSA were immobilized on a CM5 sensor chip (GE Healthcare Life Sciences, BR100530, Uppsala, Sweden) using an Amine coupling kit (GE Healthcare Life Sciences, BR100050, Uppsala, Sweden). Different concentrations of purified IRCR201 were injected into immobilized human and mouse c-Met to determine K_D_ values. To obtain the kinetic and affinity constants of IRCR201 to human and mouse c-Met, Biacore™ T100 evaluation software (GE Healthcare Life Sciences, Uppsala, Sweden) was used.

### 4.7. Epitope Mapping

The epitope mapping of IRCR201 to c-Met was performed using the scFv format of IRCR201. The scFv of IRCR201 was produced using TOP10F' *Escherichia coli* (*E. coli*) (Invitrogen, C303003, Carlsbad, CA, USA) and purified from the bacterial lysates by immobilized metal affinity chromatography (IMAC) using nickel–nitrilotriacetic acid (Ni-NTA) agarose (QIAGEN, 30210, Hilden, Germany) according to the instructions of the manufacturer. The c-Met 15-mer peptides corresponding to 1-932 of the c-Met extracellular domain were synthesized at 5 nmol/spot and covalently bound to a Whatman^®^ 50 cellulose support by the C-terminus (JPT Peptide Technologies GmbH, Berlin, Germany). The membrane of c-Met 15-mer peptides was blocked with 5% skim milk for 3 h at room temperature with shaking. The membrane was incubated with the scFv of IRCR201 in 5% skim milk at 4 °C overnight. The scFv of IRCR201 bound to the c-Met 15-mer peptides was transferred to a polyvinylidene difluoride (PVDF) membrane (Millipore, IPVH00010, Billerica, MA, USA) using the electroblotting method. The transferred PVDF membranes were washed in 1× TBST (tris-buffered saline with Tween 20) twice for 10 min. The PVDF membrane was blocked with 5% skim milk for 3 h and incubated with anti-HA-peroxidase (Roche Diagnostics, 12013819001, Mannheim, Germany) for 2 h with agitation. The membrane was washed three times with 1× TBST for 5 min and incubated with enhanced chemiluminescence (ECL) detection reagents (GE Healthcare Life Sciences, RPN2106, Little Chalfont, UK) for about 1 min. A sheet of film on the membrane was placed into a film cassette in a darkroom, according to the manufacturer’s instructions. The intensity of the bands was quantified by Multi Gauge software V3.0 (Fuji photo film Co., Ltd., Tokyo, Japan).

### 4.8. Protein Modeling and Docking

The sequences of the V_H_ and V_L_ segment of IRCR201 were assembled in the scFv format by homology modeling using the Rosetta-based computational homology modeling technique [31]. The IRCR201/c-Met docking model was made using the ZDOCK server [32]. Structure models were analyzed using PyMOL (DeLano Scientific LLC, Palo Alto, CA, USA).

### 4.9. Agonism Analysis

Wells of 96-well tissue culture plates (Costar #3595, Corning, NY, USA) were seeded with 5000 Caki1 cells in RPMI1640 medium supplemented with 10% (*v*/*v*) FBS. After culture for 24 h, cells were starved in serum-free RPMI1640 medium for another 24 h. The cells were then cultured in the presence of anti-c-Met antibodies and HGF in serum-free medium for 30 min at 37 °C. Next, the medium was removed and the cells were washed once with 1× PBS (pH 7.4). Cells were lysed and p-Akt levels were quantified by PathScan^®^ phospho-Akt1 (Ser473) chemiluminescent sandwich ELISA kit (Cell Signaling Technology, #7134, Danvers, MA, USA) according to the manufacturer’s protocol.

To clarify agonistic activity of IRCR201, Caki1 cells were starved in serum-free RPMI 1640 medium for 24 h and then treated with HGF or c-Met antibodies for 30 min. Cells were lysed and then the Western blotting experiments were proceeded.

### 4.10. c-Met Degradation Assay

U87MG, A549, and MKN45 cells were treated with 100 nM of IRCR201 for 0 min, 15 min, 30 min, 60 min, 120 min, and 240 min, and then detached by mild trypsinization, fixed using 4% paraformaldehyde solution (Biosesang, P2031, Seongnam, Korea) at 4 °C, and resuspended at a concentration of 5 × 10^5^ cells/mL in flow cytometry buffer (1% (*w*/*v*) FBS, 0.03% (*w*/*v*) NaN_3_, and 25 mM HEPES in 1× PBS, pH 7.4). To confirm the expression of c-Met on the cell surface depending on IRCR201 treatment times, fixed cells were incubated with 1:50 Met (D1C2) XP^®^ Rabbit mAb conjugated Alexa Fluor^®^ 488 (Cell Signaling Technology, #8494, Danvers, MA, USA). Cells were run on a FACSAria^TM^ III (BD Biosciences, Mountainview, CA, USA) and analyzed using the CellQuest™ program (BD Biosciences, Mountainview, CA, USA).

To analyze the total c-Met degradation of c-Met-expressing cells by IRCR201, an ELISA-based quantification method was used. When U87MG, A549, and MKN45 cells in 96-well cell culture plates (Costar, #3595, Corning, NY, USA) reached approximately 70% confluency, cells were treated with 100 nM of IRCR201 for 0 min, 15 min, 30 min, 60 min, 90 min, and 120 min. After washing with 1× PBS (pH 7.4), cells were resuspended with lysis buffer (Roche Diagnostics, 04719956001, Mannheim, Germany) supplemented with protease inhibitor cocktail tablets (Roche Diagnostics, 04719956001, Mannheim, Germany) and phosphatase inhibitor (Roche Diagnostics, 4906845001, Mannheim, Germany). The changes in c-Met protein were analyzed in a timely manner by a human HGFR/c-MET DuoSet^®^ ELISA kit (R&D systems, DY358, Minneapolis, MN, USA) according to the manufacturer’s protocol.

### 4.11. Proliferation Assay

MCF7, U87MG, A549, and MKN45 cells were seeded into 96-well white plates (Costar, #3610, Corning, NY, USA) at a density of 3,000 cells/well and cultured for 72 h with 0–100 nM of IRCR201, huOA5D5.v2, or human IgG control at 37 °C in a humidified 5% CO_2_ atmosphere. The number of viable cells was estimated by a CellTiter-Glo^®^ luminescent cell viability assay kit (Promega, G7573, Madison, WI, USA). The luminescent signal intensity of the plates was read by an Infinite^®^ m200 pro (Tecan, Männedorf, Switzerland) and normalized to the untreated group.

### 4.12. Caspase 3/7 Activity Assay

The caspase 3/7 activity was detected using the Caspase-Glo^®^ 3/7 Luciferase assay kit (Promega, G8091, Madison, WI, USA) in accordance with the manufacturer's recommendations. Cells in appropriate complete medium were incubated for 24 h with the untreated group, Human IgG, or IRCR201. After the addition of Caspase-Glo^®^ 3/7 reagent, the luminescent signal intensity was measured by an Infinite^®^ m200 pro (Tecan, Männedorf, Switzerland) and normalized to the untreated group.

### 4.13. Western Blot Analysis

When U87MG, A549, and MKN45 cells reached 70% confluency in six-well tissue culture plates (Nunc, 140675, Roskilde, Denmark), cells were incubated with IRCR201 (100 nM) or PBS in full-serum media for 24 h at 37 °C in a humidified 5% CO_2_ atmosphere. In the case of A549, conditions for treatment with HGF (50 ng/mL) were added. Cells were then lysed with Lysis-M reagent (Roche Diagnostics, 04719956001, Mannheim, Germany) supplemented with protease inhibitor cocktail tablets (Roche Diagnostics, 04719956001, Mannheim, Germany) and phosphatase inhibitor (Roche Diagnostics, 4906845001, Mannheim, Germany). Samples were denatured with 4× lithium dodecyl sulfate (LDS) sample buffer (Invitrogen, NP0007, Carlsbad, CA, USA) and 10× reducing reagent (Invitrogen, NP0009, Carlsbad, CA, USA) at 70 °C for 10 min. For Western blotting, cell lysates were resolved by sodium dodecyl sulfate-polyacrylamide gel electrophoresis (SDS-PAGE) using 4–20% Mini-PROTEAN^®^ TGX™ gels (Bio-Rad, #4561094 or #4561096, Hercules, CA, USA) and transferred to nitrocellulose membranes (Bio-Rad, #1704158, Hercules, CA, USA) using a Trans-Blot^®^ Turbo™ transfer system (Bio-Rad, Hercules, CA, USA). Blots were blocked with 5% BSA solution for 1 h at room temperature and then incubated overnight with appropriate primary antibodies at 4 °C with agitation. Primary antibodies used include the Met (D1C2) XP^®^ Rabbit mAb (Cell Signaling Technology, #8198, Danvers, MA, USA), Phospho-Met (Tyr1234/1235) (D26) XP^®^ Rabbit mAb (Cell Signaling Technology, #3077, Danvers, MA, USA), Akt Rabbit mAb (Cell Signaling Technology, #9272, Danvers, MA, USA), Phospho-Akt (Ser473) (D9E) XP^®^ Rabbit mAb (Cell Signaling Technology, #4060, Danvers, MA, USA), p44/42 MAPK (Erk1/2) Rabbit mAb (Cell Signaling Technology, #9102, Danvers, MA, USA), Phospho-p44/42 MAPK (Erk1/2) (Thr202/Tyr204) Rabbit mAb (Cell Signaling Technology, #4370, Danvers, MA, USA), and Actin (13E5) Rabbit mAb (Cell Signaling Technology, #4970, Danvers, MA, USA), all used according to the manufacturer’s recommendations. Following overnight incubation with primary antibodies, blots were washed three times with 1× PBST for 20 min, and then incubated with 1:2000 goat anti-rabbit IgG-HRP (Abcam, ab6721) as a secondary antibody for 1 h at room temperature. Blots were then washed three times with 1× PBST for 20 min, and immune-reactive bands were visualized using ECL Western blotting detection reagents (GE Healthcare Life Sciences, RPN2106 or RPN2232, Little Chalfont, UK) and scanned by ImageQuant™ LAS 4000 (GE Healthcare Life Sciences, Uppsala, Sweden).

To confirm the degradation mechanism of c-Met in A549, 100 nM concanamycin A (Abcam, ab144227, Cambridge, UK) or 5 μM MG132 (Selleckchem, S2619, Houston, TX, USA)/5 μM lactacystin (Abcam, ab141411, Cambridge, UK) mixture was pre-treated for 2 h, and IRCR201 or 5D5 was treated for 4 h at 37 °C in a humidified 5% CO_2_ atmosphere. Cells were lysed, and the Western blotting steps were conducted as described above.

### 4.14. Laser Scanning Microscopy

MKN45 cells were cultured on eight-well chamber 15 µ-slide (ibidi GmbH, #80826, Planegg, Germany). MKN45 cells were incubated with 10 µg/mL IRCR201 at 37 °C in a humidified 5% CO_2_ atmosphere. After being washed with 1× PBS (pH 7.4), the cells were fixed by 4% paraformaldehyde (Biosesang, P2031, Seongnam, Korea), permeabilized with 0.1% Triton™ X-100 (Sigma-Aldrich, X100, St. Louis, MO, USA), and blocked by adding 1% BSA. IRCR201 binding to c-Met were stained by anti-Human IgG secondary antibody conjugated Alexa Fluor^®^ 647 (Invitrogen, A-21445, Carlsbad, CA, USA), and LAMP1 was detected by anti-LAMP1 antibody conjugated Alexa Fluor^®^ 488 (Abcam, ab187591, Cambridge, UK). MKN45 cells stained fluorescence dyes were mounted with VECTASHIELD^®^ Mounting Medium with 4′,6-diamidino-2-phenylindole (DAPI; Vector Laboratories, H-1200, Burlingame, CA, USA). All fluorescence images were obtained by LSM780 (Zeiss, Jena, Germany) and analyzed by ZEN2012 (Zeiss, Jena, Germany).

### 4.15. In Vivo Therapeutic Efficacy

The efficacy of IRCR201 was evaluated in various tumor xenograft models. A549 and MKN45 single cells suspended in Hank’s Balanced Salt Solution (HBSS; Gibco, 24020117, Carlsbad, CA, USA) with Matrigel^®^ (Corning, 354234, Lowell, MA, USA) were subcutaneously injected into the right flank region of female BALB/c-nu mice (1 × 10^6^ cells per mouse in 100 μL). When the mean tumor volume reached approximately 150 mm^3^ (day 0), animals were randomized according to tumor volume to minimize intragroup and intergroup variation. After regrouping, IRCR201, huOA5D5.v2, or vehicle was intravenously administered twice per week. Each treatment group consisted of six mice. Tumor volumes were measured using three-dimensional calipers. Body weights were measured as general confirmation of toxicity.

### 4.16. Immunohistochemistry

For the immunostaining of Ki-67, c-Met, and phospho-c-Met, phospho-Akt, phospho-Erk1/2, and PECAM1, antigen retrieval was processed in formalin-fixed and subsequently paraffin-embedded MKN45 tumor tissues. After blocking with 5% bovine serum albumin in 1× PBS (pH 7.4) for 1 h at room temperature, the sections were incubated overnight with primary antibodies at 4 °C. Primary antibodies used include Ki-67 (D2H10) Rabbit mAb (Cell Signaling Technology, #9027, Danvers, MA, USA), Met (D1C2) XP^®^ Rabbit mAb (Cell Signaling Technology, #8198, Danvers, MA, USA), Phospho-Met (Tyr1234/1235) (D26) XP^®^ Rabbit mAb (Cell Signaling Technology, #3077, Danvers, MA, USA), Phospho-Akt (Ser473) (D9E) XP^®^ Rabbit mAb (Cell Signaling Technology, #4060, Danvers, MA, USA), Phospho-p44/42 MAPK (Erk1/2) (Thr202/Tyr204) Rabbit mAb (Cell Signaling Technology, #4370, Danvers, MA, USA), and PECAM1 (D8V9E) XP^®^ Rabbit mAb (Cell Signaling Technology, #77699, Danvers, MA, USA); all were used according to the manufacturer’s recommendations. After slides were washed twice in 1× PBST, tumor sections were incubated with 1:200 biotinylated anti-rabbit IgG (Vector Laboratories, BA-1000, Burlingame, CA, USA). Detection and visualization of the tumor sections were performed using the avidin/biotin/peroxidase complex (Vector Laboratories, PK-4000, Burlingame, CA, USA) and 3,3′-diaminobenzidine (Invitrogen, 750118, Carlsbad, CA, USA). For TUNEL assay, the ApopTag^®^ Peroxidase In Situ Apoptosis Detection Kit (Millipore, S7100, Billerica, MA, USA) was used according to the manufacturer’s protocol. The IHC images of the tumor sections were obtained using the Aperio imaging system (Leica Biosystems, Wetzlar, Germany).

### 4.17. Statistical Analysis

All data were analyzed with GraphPad Prism^®^ V5.01 (GraphPad Software, Inc., La Jolla, CA, USA) and expressed as the mean ± SEM unless otherwise noted. The statistical significance in tumor growth between different groups was analyzed by one-tailed two sample *t*-test.

## 5. Conclusions

IRCR201 exhibits tumor growth inhibitory activity in various cancer types harboring HGF-dependent or HGF-independent/c-Met-amplified activation.

## Figures and Tables

**Table 1:** Kinetic constants and binding affinities of IRCR201.

Proteins	K_a_ (1/Ms)	K_d_ (1/s)	K_D_ (nM)
Human c-Met ECD-Fc	4.226 × 10^6^	3.045 × 10^−3^	0.7207
Mouse c-Met ECD-Fc	3.826 × 10^6^	3.232 × 10^−3^	0.8448

K_a_ and K_d_ were measured by a Biacore™ T100 using purified IRCR201, and K_D_ was calculated by Biacore™ evaluation software. Abbreviations: K_a_, association rate constant; K_d_, dissociation rate constant; K_D_, equilibrium dissociation constant.

**Table 2:** Summary of epitope mapping.

Spot	Peptide Sequence	Amino Acid Positions	Binding Activity
C-7	LNGLGCRHFQSCSQC	515–529	−
D-7	GCRHFQSCSQCLSAP	519–533	−
E-7	FQSCSQCLSAPPFVQ	523–537	+++
F-7	SQCLSAPPFVQCGWC	527–541	++
G-7	SAPPFVQCGWCHDKC	531–545	+
H-7	FVQCGWCHDKCVRSE	535–549	−
I-7	GWCHDKCVRSEECLS	539–553	−

Epitope: 531-SAPPFVQ-537 (plexin-semaphorin-integrin (PSI) domain).

## Abbreviations

ADC	Antibody-drug conjugates
ADCC	Antibody-dependent cell cytotoxicity
ATCC	American type culture collection
BSA	Bovine serum albumin
CDR	Complementarity determining regions
DAPI	4′,6-diamidino-2-phenylindole
ECD	Extracellular domain
EGFR	Epidermal growth factor receptor
ELISA	Enzyme-linked immunosorbent assay
EMEM	Eagle’s Minimum Essential Medium
FBS	Fetal bovine serum
HBSS	Hank’s balanced salt solution
HEPES	4-(2-Hydroxyethyl)-1-piperazineethanesulfonic acid
HGF	Hepatocyte growth factor
HGFR	Hepatocyte growth factor receptor
HRP	Horseradish Peroxidase
IgG1	Immunoglobulin G1
IHC	Immunohistochemistry
IMAC	Immobilized metal affinity chromatography
IPT	Immunoglobulin–plexin–transcription
LAMP1	Lysosomal-associated membrane protein 1
MET	Mesenchymal-epithelial transition
MFI	Mean fluorescence intensity
Ni-NTA	Nickel–nitrilotriacetic acid
NSCLC	Non-small cell lung cancer
PBS	Phosphate-buffered saline
PBST	Phosphate-buffered saline with Tween 20
PECAM1	Platelet endothelial cell adhesion molecule 1
PSI	Plexin–semaphorin–integrin
PVDF	Polyvinylidene difluoride
RPMI	Roswell Park Memorial Institute
RON	Recepteur d’origine nantais
RTK	Receptor tyrosine kinase
scFv	Single-chain variable fragment
SEM	Standard error of mean
SPR	Surface plasmon resonance
TBST	Tris-buffered saline with Tween 20
TMB	3,3′,5,5′-Tetramethylbenzidine
TUNEL	Terminal deoxynucleotidyl transferase dUTP nick end labeling
VEGF	Vascular endothelial growth factor
